# Genome-Wide Analysis of the AP2/ERF Gene Family in Physic Nut and Overexpression of the *JcERF011* Gene in Rice Increased Its Sensitivity to Salinity Stress

**DOI:** 10.1371/journal.pone.0150879

**Published:** 2016-03-04

**Authors:** Yuehui Tang, Shanshan Qin, Yali Guo, Yanbo Chen, Pingzhi Wu, Yaping Chen, Meiru Li, Huawu Jiang, Guojiang Wu

**Affiliations:** 1 Key Laboratory of Plant Resources Conservation and Sustainable Utilization, South China Botanical Garden, Chinese Academy of Sciences, Guangzhou, 510650, PR China; 2 University of Chinese Academy of Sciences, Beijing, 100049, PR China; Institute of Genetics and Developmental Biology, Chinese Academy of Sciences, CHINA

## Abstract

The AP2/ERF transcription factors play crucial roles in plant growth, development and responses to biotic and abiotic stresses. A total of 119 AP2/ERF genes (*JcAP2/ERFs*) have been identified in the physic nut genome; they include 16 AP2, 4 RAV, 1 Soloist, and 98 ERF genes. Phylogenetic analysis indicated that physic nut AP2 genes could be divided into 3 subgroups, while ERF genes could be classed into 11 groups or 43 subgroups. The AP2/ERF genes are non-randomly distributed across the 11 linkage groups of the physic nut genome and retain many duplicates which arose from ancient duplication events. The expression patterns of several *JcAP2/ERF* duplicates in the physic nut showed differences among four tissues (root, stem, leaf, and seed), and 38 *JcAP2/ERF* genes responded to at least one abiotic stressor (drought, salinity, phosphate starvation, and nitrogen starvation) in leaves and/or roots according to analysis of digital gene expression tag data. The expression of *JcERF011* was downregulated by salinity stress in physic nut roots. Overexpression of the *JcERF011* gene in rice plants increased its sensitivity to salinity stress. The increased expression levels of several salt tolerance-related genes were impaired in the *JcERF011*-overexpressing plants under salinity stress.

## Introduction

The APETALA2/ethylene response factor (AP2/ERF) superfamily of transcription factors is defined by the AP2/ERF domain, which consists of about 60 to 70 amino acids and is involved in DNA binding [1–3]. On the basis of the protein structure and sequence similarity of the AP2/ERF domains, the AP2/ERF superfamily has been divided into 4 families: AP2, ERF, RAV, and Soloist [4, 5]. The AP2 family proteins contain two repeated AP2/ERF domains, the ERF and Soloist family proteins contain a single AP2/ERF domain, and the RAV family proteins contain a single AP2/ERF domain and an additional DNA-binding domain, B3. It has been hypothesized that the AP2/ERF proteins evolved from HNH-AP2 endonucleases which may have moved horizontally into plants through endosymbiosis of a cyanobacterium, viral infection, or other lateral gene transfer events [5]. The HNH-AP2 endonucleases may have spread in the genome via transposition and homing processes. Some of them may have diverged, losing the HNH domain but retaining the AP2 domain, and potentially acquiring new functions. The gain of a B3 domain in the ancestral genes of some of the AP2/ERF subfamilies resulted in the evolution of the RAV family, while the fusion of tandem repeats in the ancestral genes of some AP2/ERF genes during the evolution of plants gave rise to the AP2 family. Independent intron evolution events probably occurred in the ancestors of the AP2, Soloist, and ERF families.

Sequencing of the complete Arabidopsis genome made it possible to study the members of the AP2/ERF superfamily in this species, and their phylogenetic relationships, in detail. In Arabidopsis, a total of 147 AP2/ERF genes have been characterized, including 18 AP2, 6 RAV and 122 ERF members of the family and one Soloist gene. The AP2 genes can be divided into two subfamilies, APETALA2 (AP2) and AINTEGUMENTA (ANT). The ERF family proteins in Arabidopsis were divided into 12 groups, A1-A6 (the DERB subfamily) and B1-B6 (the ERF subfamily) by Sakuma *et al* [6]. On the other hand, Nakano *et al* [7] divided the Arabidopsis ERF genes into 12 groups (I to X, VI-L and Xb-L) based on the structures, phylogeny, chromosomal locations and conserved motifs of the genes. As genome sequencing has been extended to a wider range of plant species, genome wide identification of AP2/ERF superfamily members has been conducted in various plants, including both dicots and monocots, such as soybean (*Glycine max* L. Merr.) [8], grape (*Vitis vinifera* L.) [9], cucumber (*Cucumis sativus* L.) [10], peach (*Prunus persica* (L.) Batsch) [11], poplar (*Populus trichocarpa*) [12], castor bean (*Ricinus communis* L.) [13], rice (*Oryza sativa* L.) [7, 14], sorghum (*Sorghum bicolor* L.) [15] *etc*.

The functions of AP2/ERF gene products have been widely studied in the model plant Arabidopsis and in other plants. The AP2 subfamily genes function mainly during flower development [16, 17], and their expression is regulated by the microRNA miR172 [18]. The ANT genes have roles in the development of stem cells and meristems or in metabolism [19, 20]. RAV genes have been characterized as regulators of plant development and abiotic stress responses [21, 22]. The Arabidopsis *Soloist* gene, At4g13040, encodes a positive regulator of salicylic acid accumulation and basal defense against bacterial pathogens [23]. The DREB subfamily proteins are considered to be mainly regulators of plant responses, particularly abiotic stress responses [24]. The ERF subfamily genes are considered to participate in biotic and abiotic stress responses [25, 26], the oxygen sensing pathway [27, 28], nutrition signaling pathways [29, 30] and plant hormone signaling pathways [31–36], and also in regulating plant metabolism [37, 38], growth and development [36, 39, 40].

Physic nut (*Jatropha curcas* L.) is a multipurpose woody plant belonging to the Euphorbiaceae family. Its abilities to endure drought and adapt easily to barren soil, and the high oil content of its tree-borne seeds, mean that the physic nut has emerged as a source of biofuel [41–43]. Our previous study identified a total of 117 putative AP2/ERF genes (*JcAP2/ERF*) in the physic nut genome [44]. In this study, we attempted to establish a more comprehensive picture of the *JcAP2/ERF* gene superfamily in physic nut. First, we investigated the JcAP2/ERF gene superfamily in detail. The data we obtained revealed 119 distinct AP2/ERF gene sequences in this species. Secondly, we characterized the exon—intron organization and conserved domains of these genes, then subjected them to phylogenetic analysis. Thirdly, we analyzed the expression of the physic nut *JcAP2/ERF* genes under normal growing conditions and when exposed to various abiotic stresses. Finally, we analyzed the functions of *JcERF*0*11* by overexpressing it in Arabidopsis and rice, the model plants for dicots and monocots, and found that it result in different biological effects in transgenic plants of the two species.

## Materials and Methods

### Sequence database searches

Sequences of AP2/ERF domain-containing proteins from Arabidopsis were downloaded from the Arabidopsis genome sequence, TAIR 9.0 release (http://www.Arabidopsis.org/), while sequences for rice, castor bean, *Chlamydomonas reinhardtii*, and *Physcomitrella patens* were downloaded from Phytozome (http://phytozome.jgi.doe.gov/pz/portal.html) and sequences for grapevine were downloaded from Licausi *et al* [9].

We searched for AP2/ERF domain-containing genes in the physic nut (*J*. *curcas* L.) genome database of the Kazusa DNA Research Institute (http://www.kazusa.or.jp/jatropha/) [45] and in our own genome database (available from DDBJ/EMBL/GenBank under the accession number AFEW00000000) [44]. To search for AP2/ERF domain-containing genes in the physic nut, we used Arabidopsis AP2/ERF proteins from each subgroup as query sequences for tBlastn and Blastp searches against the physic nut genome sequences and against predicted protein sequences. Next, we corrected errors in the annotation of AP2/ERF coding domain sequences on the basis of the physic nut EST database available from GenBank (http://www.ncbi.nlm.nih.gov/), and our own physic nut and *J*. *integerrima* EST datasets (accessions SRA197144 and SRA197148 in GenBank). The exon—intron structures of *AP2/ERF* genes were determined by comparing the coding sequences and the corresponding genomic sequences using the Gene Structure Display Server (http://gsds.cbi.pku.edu.cn/). Chromosomal positions of the *AP2/ERF* genes were mapped onto the physic nut linkage map [44].

### Phylogenetic tree construction

Multiple sequence alignments of the conserved AP2/ERF domain sequences were performed using Clustal W2 (http://www.ebi.ac.uk/Tools/msa/clustalw2/). Unrooted maximum-likelihood (ML) trees were constructed using the LG model with approximate likelihood ratio test (aLRT) SH-like branch support steps in PhyML version 3.0 (http://atgc.lirmm.fr/phyml/) [46], and the results were displayed with the Mega software package version 5 [47].

### Gene cloning and plant transformation

Total RNA was extracted from physic nut leaves and first-strand cDNA was synthesized according to Xiong *et al* [48]. Fragments containing the complete coding domain sequences of physic nut ERF genes (*JcERF011*) was amplified with the primer pairs listed in S1 Table. The PCR products were cloned into the pMD 18-T vector (TaKaRa, Otsu, Japan) and subjected to DNA sequencing. The resulting fragments were digested using the restriction enzymes *Sac* I and *Xba* I and inserted into the corresponding restriction sites of the pCAMBIA1301 vector under the control of the CaMV35S promoter. *Agrobacterium* lines harboring the constructs were transformed into Arabidopsis (Col-0 ecotype) [49] and rice (*japonica cv*. Zhonghua 11) [50]. Single-insertion homozygous transgenic lines were chosen for the next stage of analysis by testing the expression of the reporter gene β-glucuronidase of in T2 plants.

### Preparation of physic nut materials

Seeds of the inbred physic nut cultivar GZQX0401 were sterilized with KMnO_4_ solutions (1/5000) for 30 min, leaved in distilled water for 12 h, and then planted in sand. When cotyledons of the germinated seeds were fully expanded, seedlings were transferred to a 3:1 mixture of sand and soil in a greenhouse (30–35°C) in Guangzhou (113.3°E, 23.1°N) illuminated with natural sunlight. After emergence of the first true leaf, the trays were irrigated with 1 L Hoagland nutrient solution (pH 6.0) once every two days at dusk. Samples of the roots, stem cortex, and leaves collected at the six-leaf stage and seed samples (early development (S1; 14 days after pollination) and the filling (S2; 41 days after pollination) stages), were frozen immediately in liquid nitrogen and stored at -80°C prior to digital gene expression analysis.

The Pi and N deficiency treatments were begun at the six-leaf stage (eight weeks after germination), after removing most nutrients by five washes with 1 liter of tap water. The plants assigned to the P- or N- deficiency treatments were then irrigated daily with Hoagland nutrient solution minus phosphorus or minus nitrogen). For the salinity treatment, the seedlings were irrigated with Hoagland solution plus 100 mM NaCl every day. For the drought treatment, irrigation was withheld. Roots were sampled after 1 day, 2 days and 4 days of drought stress; after 2 hours, 1 day and 4 days of salinity stress; and after 2 hours, 1 day and 4 days of phosphorus and nitrogen deficiency. Samples were frozen immediately in liquid nitrogen and stored at -80°C prior to quantitative PCR (qRT-PCR) analysis.

### Arabidopsis growth conditions

Transgenic and wild-type seeds were surface-sterilized and incubated in the dark at 4°C for 2 days. For the purpose of harvesting seeds, plants were grown in pots containing a 1:1 mixture of vermiculite and peat moss of similar density under a long-day photoperiod (16 h light/8 h dark) at 22 ± 2°C in a growth chamber.

### Rice growth conditions and treatments

After germination, seedlings were cultured in Yoshida’s culture solution [51] at 25°C under 16/8 h (light/dark) conditions in a growth chamber. Two weeks later, seedlings were planted in soil in plastic pots and grown in a greenhouse under natural sunlight. For RNA isolation, leaves from two-week seedlings were sampled. For the salt tolerance test, germinated seeds (shoots were cv. 0.5 cm in length) were transferred to absorbent cotton infiltrated with Yoshida’s culture solution containing 0 mM (control), 150 mM or 200 mM NaCl in glass bottles. This test was repeated 3 times with 3 biological repeats for each test. For RNA isolation, shoots were sampled after 5 days of growth under normal and salt conditions.

### Electrolyte leakage test

After washed three times using deionized water, leaves (0.15 g) were put in a test tube containing 9 mL of deionized water. The leaf samples were immersed and vibrated occasionally at 25°C for 2 h, and then the electrical conductivity of the solution (A1) was measured using a conductivity meter. After boiling the samples for 10 min, their conductivity (A2) was measured again after the solution was cooled to room temperature. The relative electrical leakage (REL) was calculated as follows: REL (%) = A1/A2×100.

### RNA isolation and expression analysis

For rice leaves, total RNA was isolated using Trizol^®^ reagent (Invitrogen, http://www.thermofisher.com), following the manufacturer’s instructions. For physic nut roots, stem cortex, leaves and seeds, total RNA was extracted from using Hipure plant RNA mini kit (Promega, http://cn.promega.com/) according to the manufacturer’s instructions. The first-strand cDNA was synthesized from 3 μg samples of the total RNA, using M-MLV reverse transcriptase (Promega) according to the manufacturer’s instructions. Primers used in this study are listed in S1 Table. Expression levels of the physic nut ERF gene in transgenic plants were examined by semi-quantitative RT-PCR, using gene-specific primer pairs (S1 Table). The cDNA fragment of *OsUbiquitin* (S1 Table) was used as controls.

Quantitative real-time PCR was performed using a LCS480 system (Roche, http://www.roche.com/). Each 20 μL reaction volume included 10 μL of 2 × SYBR Premix ExTaq, 0.4 μL of forward and reverse primer (10 μmol), 2 μL of diluted cDNA solution, and 7.2 μL of ddH_2_O. The thermal profile used for all PCR amplifications was: 10 min at 95°C for DNA polymerase activation, followed by 40 cycles of 15 s at 95°C and 35 s at 60°C. Expression levels were calculated using the 2^-ΔΔCT^ method, with *JcActin* or *OsUbiquitin* as the reference gene for physic nut or rice, respectively. Each PCR assay was run in duplicate for three independent biological replicates.

### Statistical analysis

All experiments included three or six biological repeats, and data were analyzed with a Duncan test [52] using the SAS software package (http://www.sas.com/en_us/software/sas9.html).

## Results

### Identification of AP2/ERF genes in physic nut

**Fig 1:**
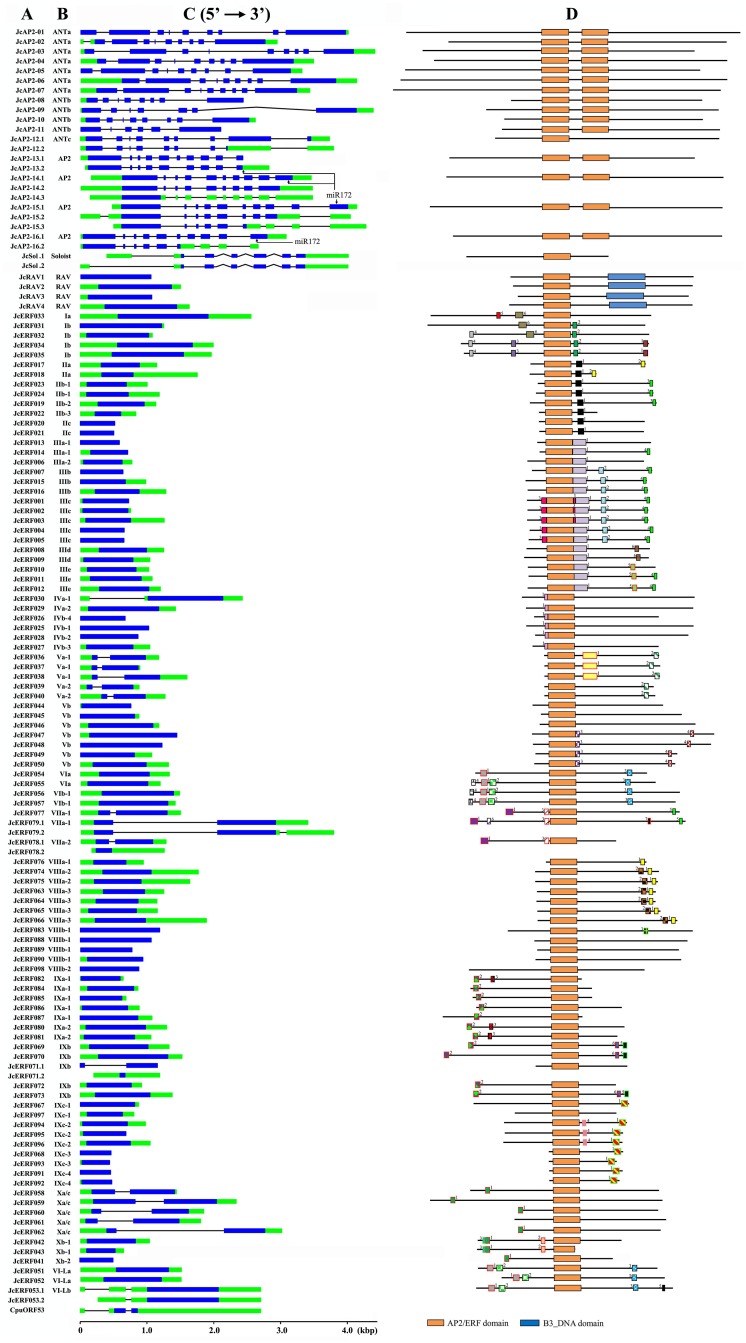
*JcAP2/ERF* gene structures and motif locations. (A), Gene name. (B), Proposed subgroup to which the gene belongs. (C), Exon/intron arrangements of *JcAP2/ERF* genes. Exons and introns are represented by boxes (open reading frames in blue and untranslated region in greens) and black lines, respectively, and their sizes are indicated by the scale at the bottom. (D), List of common motifs identified by Nakano et al [7] which are present in physic nut ERF proteins.

As result of searching for AP2-domain containing proteins, a total of 119 putative AP2/ERF genes were identified in the physic nut genome. Fifteen of these genes were assigned to the AP2 family due to their tandemly repeated double AP2/ERF domain (designated JcAP2-01 to JcAP2-11, and JcAP2-13 to JcAP2-16). Four of them (designated JcRAV1 to JcRAV4) were assigned to the RAV family since they contained a single AP2/ERF domain together with a B3 type domain. One hundred of the genes contained a single AP2/ERF domain. Within these, *JCGZ_21099* (designated *JcAP2-12*) showed the highest similarity to the Arabidopsis AP2 family gene At2g41710, while *JCGZ_17512* (designated *JcSoloist*) showed the highest similarity to the Soloist family gene At4g13040. The other 98 genes in this category were assigned to the ERF family and designated JcERF001 to JcERF098 (S2 Table). Two ERF genes, *JCGZ_01610* (*JcERF043*) and *JCGZ_13480* (*JcERF071*), were putative pseudogenes. There was a nonsense mutation within the AP2/ERF domain-encoding sequence in the *JcERF043* gene. The product of *JCGZ_13480* (*JcERF071*) had three amino acids missing from the AP2/ERF domain, and its mRNA (EST) sequence did not encode an AP2/ERF containing protein (Fig 1 and S1 Fig). Amino acid motifs located outside the DNA binding domain [7] in most physic nut ERF proteins showed sequence conservation with their Arabidopsis orthologs (Fig 1 and S2 Table).

### Phylogenetic analysis of physic nut AP2, RAV, and Soloist proteins

We inferred the phylogenetic relationships of the physic nut AP2, RAV, and Soloist proteins with proteins from the green alga *C*. *reinhardtii*, *P*. *patens*, Arabidopsis, and rice (S2A Fig), as well as all AP2/ERF proteins from physic nut, *C*. *reinhardtii*, and *P*. *patens* (S2C Fig), using the conserved AP2/ERF domains. The phylogenetic trees showed that the AP2 family had diverged into the APETALA2 (AP2) and AINTEGUMENTA (ANT) groups in higher plants. The trees also indicated that the ANT genes from physic nut could be further divided into three clades: ANTa (JcAP2-01 to JcAP2-07), ANTb (JcAP2-08 to JcAP2-11), and ANTc (JcAP2-12). ANTc proteins contained a single AP2/ERF domain. Ten genes from *P*. *patens* which each contained two AP2/ERF domains could be clearly placed in the ANT subgroup.

**Fig 2:**
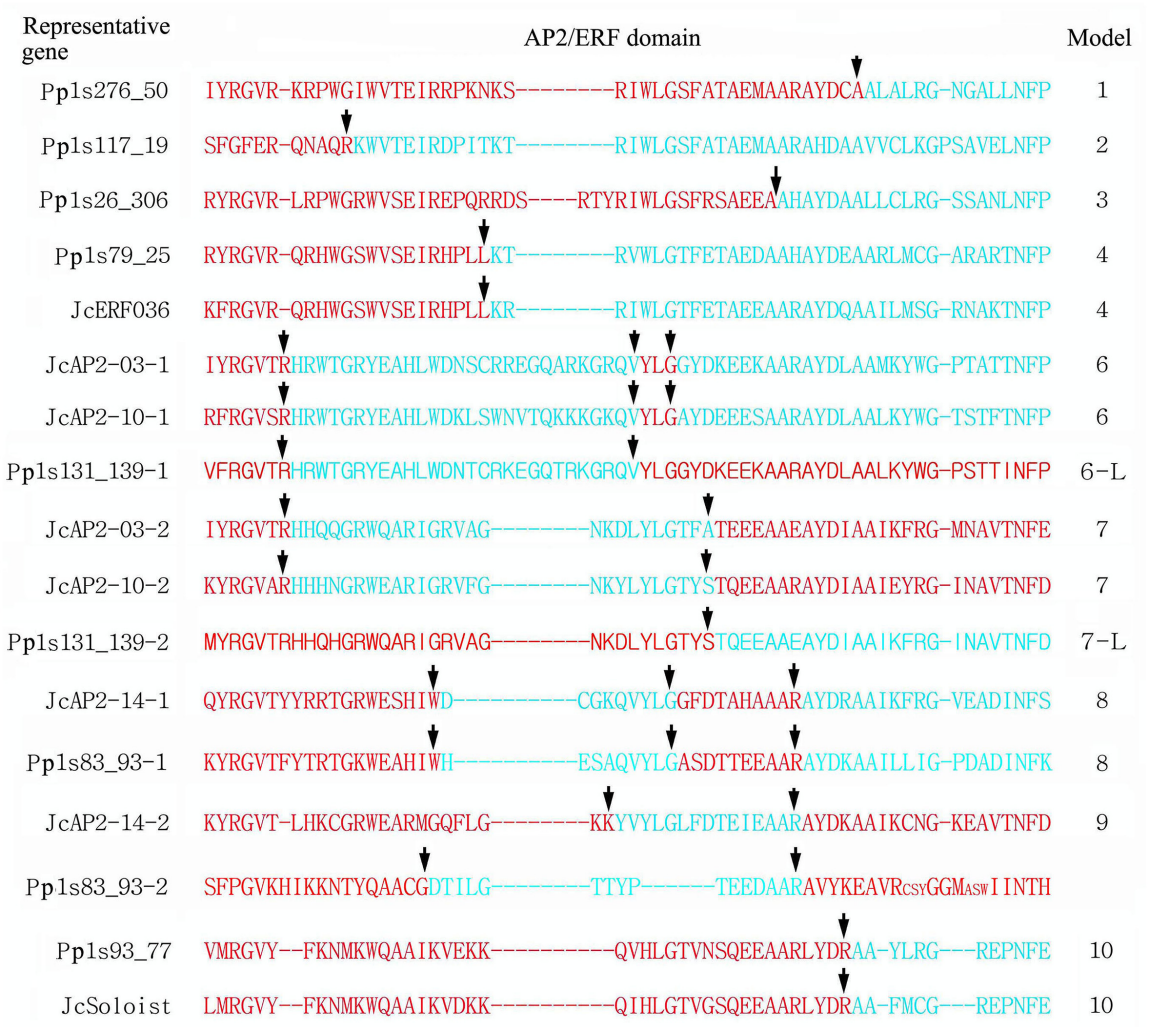
Alignment of the amino acids of the AP2/ERF domain of representative AP2/ERF genes, indicating exon‒intron structure models. Intron positions, relative to amino acid residues, are indicated by arrows. Arrows positioned between the coding sequences for two amino acids indicate that splicing occurs just before the second amino acid. Arrows pointing to amino acids indicate that splicing occurs within the amino acid coding sequences.

Arabidopsis had two more AP2 subfamily genes than physic nut, but three members of the subfamily (At2g39250, At3g54990, and At5g60120) encoded proteins containing single AP2/ERF domains. In the case of *P*. *patens*, only one sequence, Pp1s83_93V6.1, was grouped into the AP2 subfamily on the phylogenetic trees, and the R2 region of its AP2/ERF domain was divergent from those in other species (S2B Fig). Physic nut ANTa and ANTc genes contained 8 introns within their open reading frame domains (ORFs); ANTb genes mostly contained 7 introns (the fifth intron was absent in JcAP2-09, the first and the sixth introns were absent in JcAP2-11). All AP2 subfamily genes contained 9 introns. Alternative splicing of messenger RNAs was observed for all genes in the ANTc and AP2 subfamilies in physic nut (Fig 1). However, ANT genes shared the same exon-intron structure in the AP2/ERF domain coding sequences (Model 6 and 7), an organization which was different from those in AP2 subfamily genes (Model 8 and 9) (Fig 2). The four physic nut AP2 subfamily genes all contained a target site for miR172 (*JcAP2-13*: ctgcagcatcatcatgattcg; *JcAP2-14*: ctgcagcatcatcaggattcc; *JcAP2-15* and *-16*: ctgcagcatcatcaggattct) (Fig 1A) which is conserved across AP2 orthologs form higher plants [18] (Fig 1).

Physic nut had 4 RAV genes, two fewer than Arabidopsis. Physic nut, Arabidopsis and rice each had a single Soloist gene in the genome. *P*. *patens* had two RAV genes and four Soloist genes, but no genes from *C*. *reinhardtii* could be grouped into these two families. The physic nut RAV genes were intronless, while the physic nut Soloist gene contained 5 introns (Fig 1). The exon—intron structure of the AP2/ERF domain coding sequences in *Soloist* genes was conserved in physic nut and *P*. *patens* (Model 10) (Fig 2).

### Phylogenetic analysis of JcERF proteins

The ERF family proteins in Arabidopsis are divided into 12 groups, A-1 to A-6 (DREB subfamily) and B-1 to B-6 (ERF subfamily) by Sakuma et al [6]. Nakano et al [7] divide the ERF genes from Arabidopsis and rice into 12 (I to X, VI-L and Xb-L) and 15 (I to XIV, and VI-L) groups, respectively, based on the structures, phylogeny, chromosomal locations, and conserved motifs of the genes. To investigate the evolutionary relatedness of the 97 ERF genes identified in physic nut to ERF genes from Arabidopsis (122) and rice (132), we performed phylogenetic reconstruction using the conserved AP2/ERF domain. The resulting phylogenetic tree (S2D Fig) was in accordance with previous studies [6, 7] with respect to the genes from Arabidopsis in each clade. According to this phylogenetic tree, physic nut ERF genes could be divided into 11 groups or 43 subgroups followed the nomenclature proposed by Nakano et al [7] (S2D Fig and S3 Table). The DREB subfamily genes in physic nut could be classified into the proposed 4 groups (I to IV) (or six groups of A-1 to A-6, proposed by Sakuma *et al* [6] and could be further divided into 19 subgroups. JcERF026 had CMIV-1 and CMIV-3 motifs (Fig 1 and S2 Table) which were located outside the DNA binding domain [7], as did JcERF028, and was assigned to subgroup IVb. JcERF022 shared the highest amino acid sequence similarity to subgroup IIb proteins and was assigned to IIb-3. The ERF subfamily genes from physic nut could be classified into the previously-proposed 7 groups (V to X, and VIb-L) (or the 6 groups, B-1 to B-6, proposed by Sakuma et al [6] and could be further divided into 24 subgroups. JcERF041 shared the highest amino acid sequence similarity to JcERF042 and was assigned to subgroup Xb-2. Physic nut had multiple copies of genes in 28 of the 43 subgroups, but no member of subgroup Xb-L, which existed in Arabidopsis, and none in groups XI-XIV from rice. Genes in subgroups IIb-3, IVb-4, and Xb-2 did not existed in Arabidopsis or rice, but they were present in the genomes of castor bean (*Ricinus communis* L.) (S2E Fig and S3A Table) and/or *Vitis vinifera* (S2F Fig and S3A Table). A total of 36 subgroups of ERF genes were present in the observed species in this study, while 40 were present in both physic nut and Arabidopsis (S3A Table).

Upstream open reading frames (uORFs) encoding CPuORF53/54 proteins (At1g25472 and At1g68552) are present in the 5' UTR of the mature mRNAs from two Arabidopsis VI-Lb genes, At1g25470 and At1g68550 [53], and they were also found in *JcERF053* (VI-Lb) (Fig 1). Physic nut genes in six subgroups, Va-1, Va-2, VIIa-1, VIIa-2, Xa/c-1, Xa/c-2, and *JcERF071* in IXb, had an intron within their open reading frame (ORF), and in the case of genes Va-1 and Va-2 the intron was located within the AP2/ERF domain coding sequences (Fig 2). In Arabidopsis, genes in subgroups Va, VII, Xa, and Xc were also intron-containing. No intron was found in any ORF in DREB subfamily genes in physic nut, although the *JcERF030* (IVa) gene had an intron in the 5' UTR (Fig 1), whereas one Arabidopsis DREB gene, At2g40340 (IVa-3), had an intron in its ORF.

According to the phylogenetic tree constructed using the AP2/ERF proteins from physic nut, *C*. *reinhardtii*, and *P*. *patens*, no genes from *C*. *reinhardtii* were members of the DREB subfamily, while genes from *P*. *patens* were grouped into both the DREB (53) and the ERF (47) subfamily (S2C Fig and S3A Table). A total of 47 *P*. *patens ERF* genes contained introns within their ORFs. The exon—intron structures of *P*. *patens ERF* genes were classified into five types. The introns in models 1–4 were in different sites in the AP2/ERF domain coding sequences, while the intron sites in model 5 genes were out of this region (Fig 2). Most genes (29) belonged to model 1. Eighteen intron-containing genes belonged to the DREB subfamily, and of these, 15 genes corresponded to models 1–3, while 29 genes belonged to the ERF subfamily and 21 of these genes contained models 1 and 4 in *P*. *patens*. The introns in subgroup Va genes of higher plants belonged to model 4, while in other genes of higher plants belonged to model 5.

### Chromosomal locations of *JcAP2/ERF* genes

**Fig 3:**
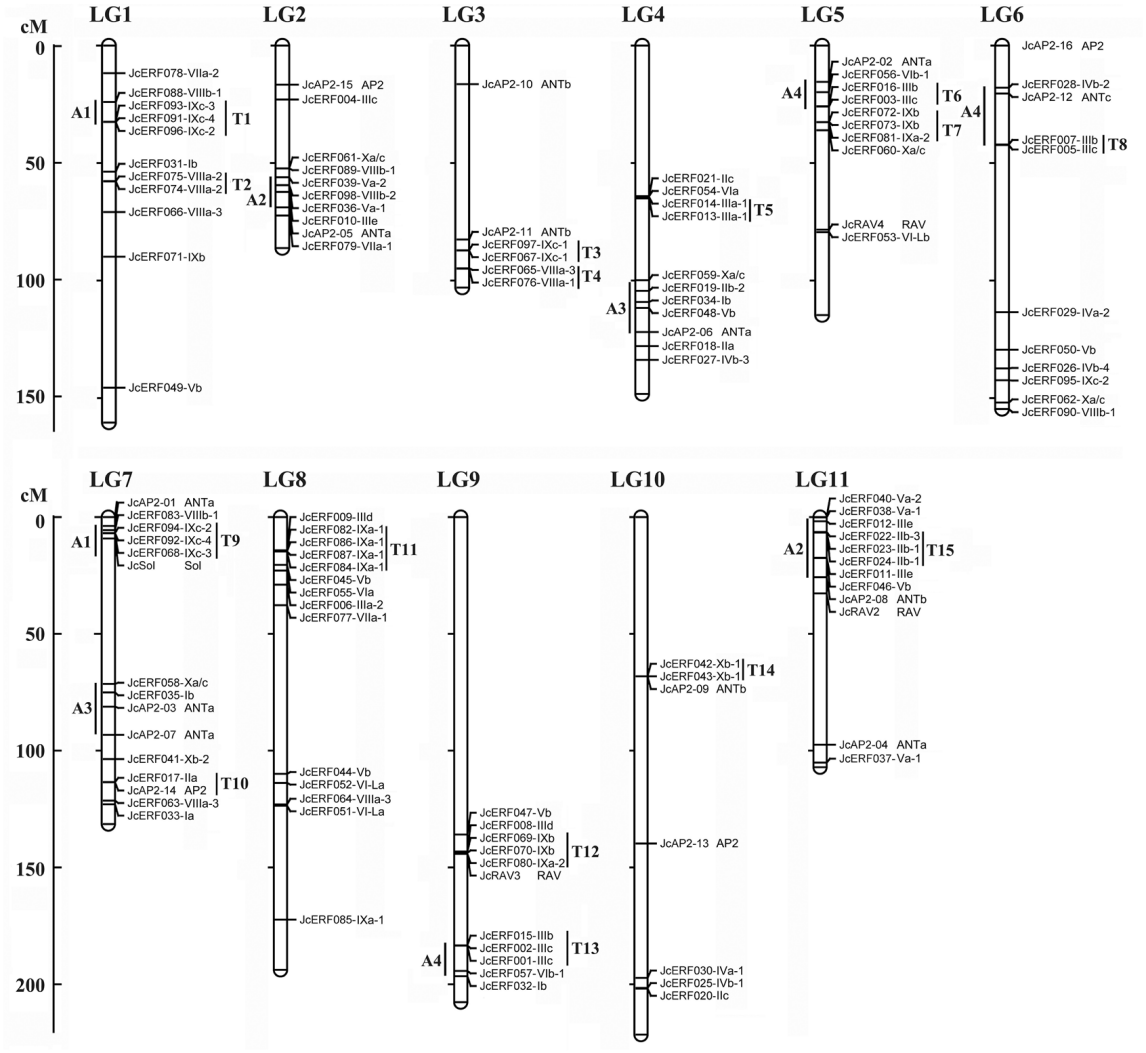
Chromosomal localization of physic nut *JcAP2/ERF* genes based on the linkage map. In total, 118 *JcAP2/ERF* genes were mapped to the 11 linkage groups (LGs). The scale is in centiMorgans (cM). T, tandem duplication; A, ancient segmental duplication identified based on genome synteny.

A total of 118 *JcAP2/ERF* genes could be mapped onto the 11 linkage groups (LGs) of physic nut [44]. These *JcAP2/ERF* genes were nonrandomly distributed on the LGs. The occurrence of segmental and tandem duplication events affecting *ERF* genes in Arabidopsis and rice has been reported previously [7]. In physic nut, four gene pairs were produced from the genome triplication undergone by ancient dicotyledons (A) (Fig 3). They were A1 on LGs 1 and 7; A2 on LGs 2 and 11; A3 on LGs 4 and 7; and A4 on LGs 5, 6, and 9 (Fig 3). Tandem duplicates, defined as tandem repeats which were located within 50 kb from each other or were separated by < 4 non-homologous spacer genes [54], were observed for the *AP2/ERF* genes in the physic nut genome. About 30.3% (N = 36) of these physic nut genes were present as tandem repeats (T) at 15 loci on 10 LGs. Genes at five tandem duplicate loci (T2, T3, T5, T11, and T14) were grouped into the same subgroup, whereas duplicate genes at other loci grouped into different subgroups on the phylogenetic trees. All of the tandem repeats in physic nut were likely to be of ancient origin because they also existed in the genomes of the other species analyzed here (S3B Table).

### Analysis of expression of *JcAP2/ERF* genes

We assessed the expression profiles of physic nut *AP2/ERF* genes in the roots, stems (stem cortex), leaves and seeds using Digital Gene Expression (DGE) tag profiling, a next-generation sequencing-based method that allows the spatial distribution of transcripts to be analyzed [44]. The plant seeds we collected allowed for study the early development (S1) and the filling and maturation (S2) stages [55]. Expressed sequence tags (ESTs) for 93 *JcAP2/ERF* genes were detected in the physic nut EST database. Another 14 *JcAP2/ERF* genes were found in the *J*. *integerrima* EST database. A further 9 *JcAP2/ERF* genes for which no ESTs were available were observed in the DGE database at low levels of expression (S4 Table). These results imply that 116 (97.5%) of the 119 *AP2/ERF* genes are expressed on the basis of the databases currently available.

**Fig 4:**
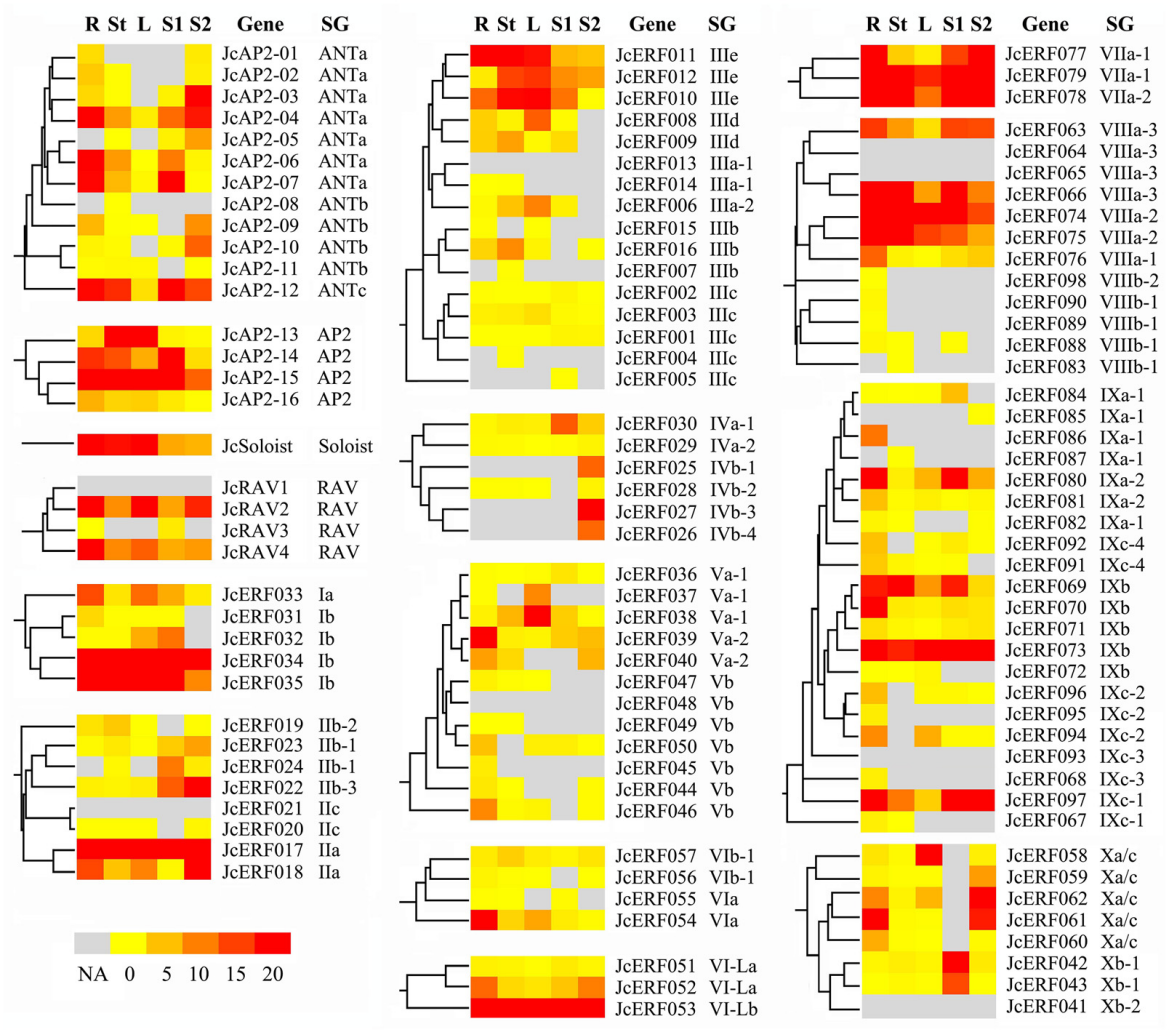
Relative levels of expression of *JcAP2/ERF* genes, divided into different subgroups. Relative expression level of each *JcAP2/ERF* gene in roots (R) and stem cortex (St) and leaves (L) (sampled from six- to ten- leaf physic nut plants) is the average value from 6 biological repeats of digital expression profile tag analysis [44]. Relative expression level of each *JcAP2/ERF* gene in seeds is the average value of 3 points of early development stage (S1) and 4 points of filling and maturation stage (S2) [55]. SG, subgroup; NA, not available.

Many of the *JcAP2/ERF* genes showed patterns of expression that varied according to tissue and developmental stage of seeds. Of the ANTa subgroup genes, *JcAP2-06 and -07* were highly expressed in roots and S1, while *JcAP2-04* was highly expressed in roots, S1, and S2. *JcAP2-03* and *JcAP2-05* were expressed at the highest level in S2. Two ANTa subgroup genes, *JcAP2-09* and *JcAP2-10*, were observed to be expressed in roots and S2 at a moderate level, and in S2 alone at a high level, respectively. The expression patterns of the four *AP2* genes also varied depending on the tissues tested. *JcAP2-15* was highly expressed in all the tissues analyzed. *JcAP2-14* was expressed much more highly in seed stage S1 than in S2. Two of the four RAV genes were observed to be expressed at high or moderate levels in all the tissues tested (Fig 4). Among the DREB/ERF genes, seven (*JcERF017*/*034*/*053*/*073/074/078/079*) were highly expressed in all tissues tested (TPM) ≥ 10), while 26 were highly expressed in roots, 15 in stems, 16 in leaves, 22 in S1, and 18 in S2. The expression levels of 8 genes (*JcERF010*/*024*/*032*/*035*/*042*/*043*/*069*/*080*) were over 5 times higher in S1 than S2, whereas the expression levels of 7 genes (*JcERF018/025*/*026*/*027*/*061/062/078*) were higher in S2 than in S1 (Fig 4 and S4 Table). Genes in 20 of the 29 multiple copy-containing ERF subgroups, including four tandem duplicates (T3, T11, T12, and T15) and five duplicates from genome triplication events (*JcERF010/011* and *JcERF036/038* in A2, *JcERF034/035* and *JcERF058/059* in A3, and *JcERF007/015/016* in A4), were differentially expressed among the tissues tested (Fig 4 and S4 Table).

In order to detect the potential roles played by *JcAP2/ERF*s in abiotic stress, we evaluated the expression of *JcAP2/ERF* genes in roots and leaves under drought [56], salinity [57], nitrogen-starvation and phosphorus-starvation stresses (unpublished data), using our next-generation sequencing-based DGE tag database; the results were reported as fold changes with respect to the controls (S4 Table). The expression levels of 38 *JcAP2/ERF* genes showed at least a 2-fold increase or decrease. Of these, six genes were expressed in response to a single treatment, while the others responded to more than one treatment (S4 Table). The number of genes expressed in response to drought, salinity, nitrogen starvation and phosphate starvation was 30, 28, 15, and 22 respectively.

**Fig 5:**
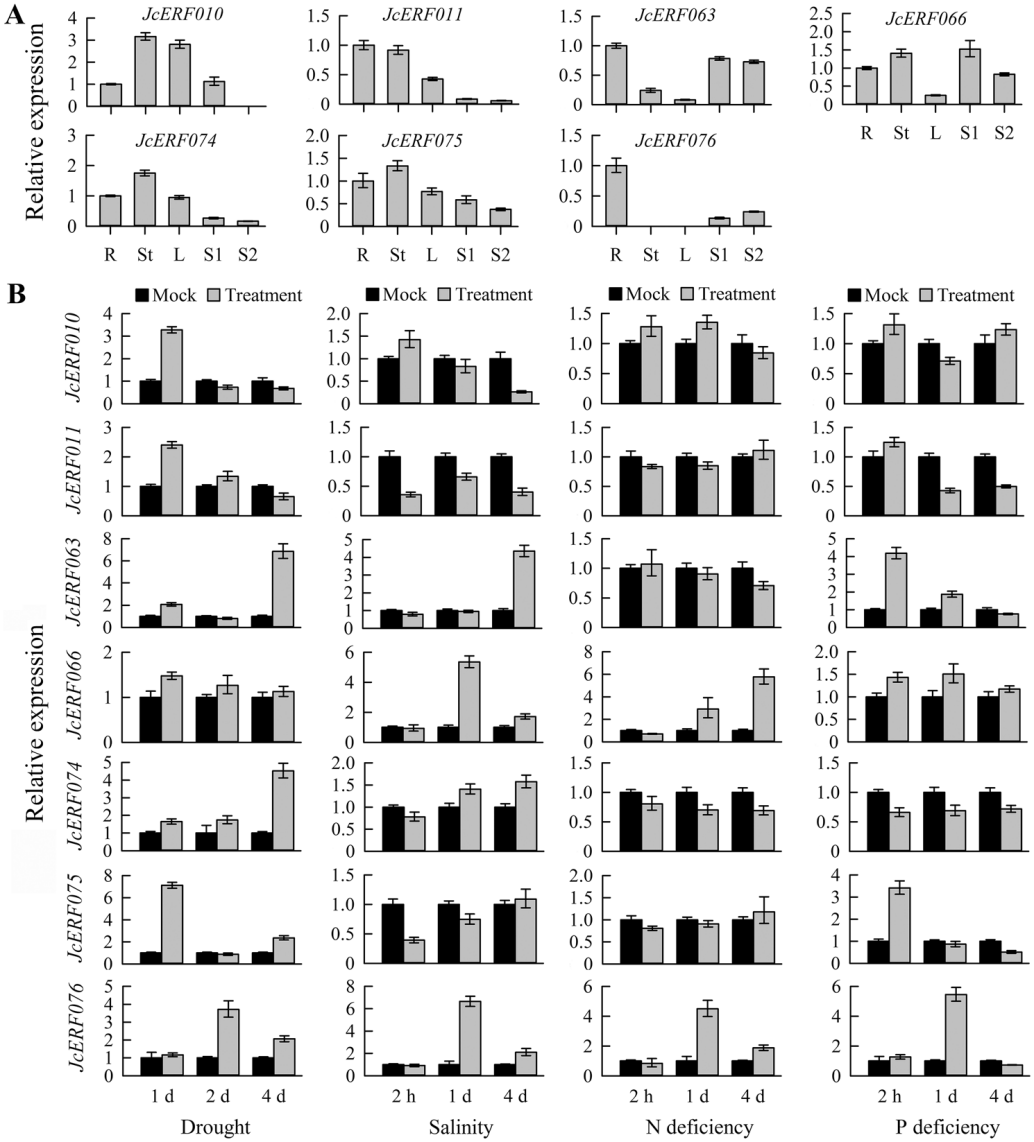
Expression analysis of selected *JcAP2/ERF* genes. (A), qRT-PCR analysis of *JcAP2/ERF* genes in roots (R), stem cortex (St), leaves (L), and seeds of 14 (S1) and 41 (S2) days after pollination. Relative expression was normalized to the reference gene *JcActin* (internal control). Bars show means ± SD of three biological replicates. (B), qRT-PCR analysis of *JcAP2/ERF* genes in roots under different abiotic stresses. The relative expression was normalized to the reference gene *JcActin* as an internal control. The bars show standard deviations of the repeats. Each assay was run in triplicate for two independent biological repeats.

To verify the DGE tag data, we examined the expression levels of 2 DREB genes (subgroup IIIe) and 5 ERF genes (subgroup VIIIa) in different tissues (Fig 5A) and in roots after onset of drought, salinity, phosphorus and nitrogen deficiency stresses (Fig 5B) by qRT-PCR analysis. The results were generally consistent with the abundance of their transcripts and expression changes observed in the DGE tag profiling experiments, suggesting that the digital expression data were generally accurate.

### Functional analysis of *JcERF011*

In functional studies on physic nut *AP2/ERF* genes, it has been reported that overexpression of *JcERF066* [58] and *JcERF034* [59] in Arabidopsis, and *JcERF079* in tobacco [60], increased the tolerance of transgenic plants to freezing and/or salt stresses. To investigate the functions of additional physic nut *AP2*/*ERF* genes, several genes that demonstrated changes in expression as a result of the stresses that we imposed were overexpressed in Arabidopsis and rice under the control of a CaMV 35S promoter. We observed that overexpression of *JcERF011* caused different biological effects in Arabidopsis and in rice.

**Fig 6:**
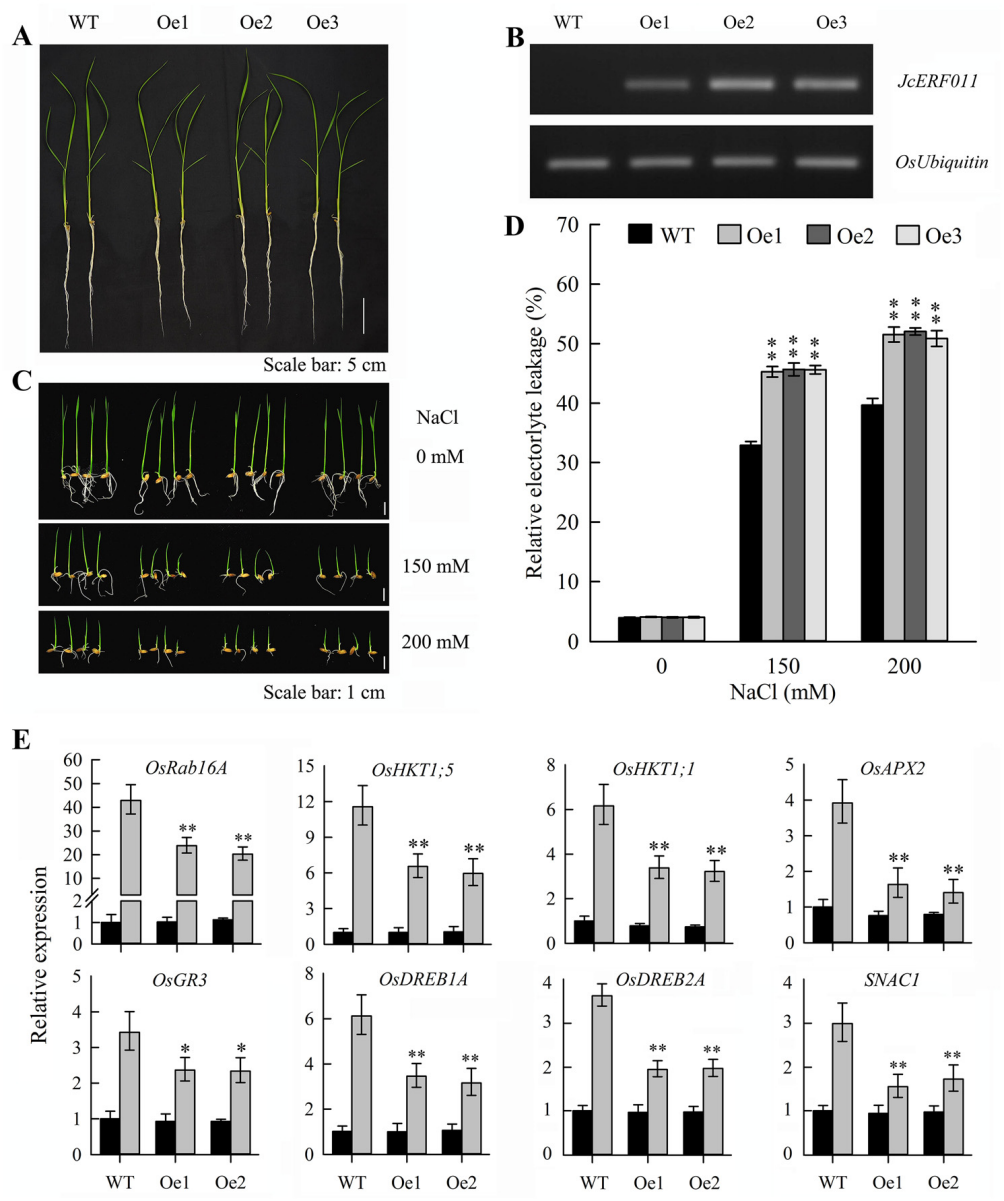
Salt stress tolerance tests on *JcERF011* overexpressing rice lines. (A), Two-week-old transgenic rice seedlings. (B), Relative levels of *JcERF011* transcript in different transgenic rice lines (Oe1, Oe2 and Oe3) determined by semi-quantitative RT-PCR. (C), Salt stress tolerance tests on *JcERF011* overexpressing rice lines. Two days after germination, the germinated seeds (shoots were about 5 mm in length) were transferred to absorbent cotton infiltrated with Yoshida’s culture solution containing 0 mM (CK), 150 mM, or 200 mM NaCl in glass bottles. Seedlings were sampled after 5 days of growth. (D), Relative electrolyte leakage from leaves. The experiment included 3 biological replicates. Values represent means of n = 15 ± SD (Duncan test: **, P < 0.01). (E), Relative expression levels of salt stress-responsive genes. The black column and grey column represent normal (Yoshida’s culture solution) and salinity stress (containing 150 mM NaCl) conditions, respectively. The experiment included 3 biological replicates, each with two technical replicates. Values represent means of n = 6 ± SD (Duncan test: *, P < 0.05; **, P < 0.01).

The gene *JcERF011* belonged to subgroup IIIe (DREB subfamily A-4), and its expression in physic nut roots was downregulated by Pi starvation and salinity stresses. Overexpression of *JcERF011* (*OeJcERF011*) in Arabidopsis resulted in severe growth inhibition under normal growing conditions (S3 Fig), and we could not obtain any seeds from the transgenic plants. In contrast, overexpression of *JcERF011* in rice did not affect plant growth and development under normal growing conditions (Fig 6A and 6C), but increased the sensitivity of rice seedlings to salinity stress. The growth of *OeJcERF011* seedlings was stronger suppressed than the wild-type seedlings under salt stress conditions (Fig 6C). The shoot heights of both wild-type and *OeJcERF011* lines were 7.7±0.6 cm when growing in normal nutrient solution, while they were 3.9±0.3 cm and 2.6±0.2 cm for wild-type and 2.7±0.4 cm and 1.7±0.3 cm for *OeJcERF011* when grown in 150 mM and 200 mM NaCl solution, respectively, for five days. The electrolyte leakage was one of the important indexes of cell membrane damage in the plant stress response. The relative electrolyte leakage from *OeJcERF011* leaves was higher than wild-type leaves after the salt stress treatments. This result indicated greater cell membrane damage of *OeJcERF011* leave cells than wild-type leave cells under these salt stress conditions (Fig 6D).

A number of salt response-genes were confirmed to be associated salt tolerance in rice. Next, we tested the expression levels of several genes which have been confirmed to be response to salinity stress in rice seedlings and overexpressing these genes could improve tolerance of the transgenic plants to salinity stress. OsRAB16A belongs to the Group 2 LEA gene family, which is consistent with their role in stabilizing cellular structures during dehydration stress [61, 62]. *OsHKT1*;*1* and *OsHKT1*;*5* genes are critical for salt tolerance through exclusion of Na^+^ ions from sensitive cells in rice [63]. *OsAPX2* and *OsGR3* genes are involved in scavenging reactive oxygen species [64, 65]. As shown in Fig 6E, the increased expression levels of *OsRAB16A*, *OsHKT1*;*1*, *OsHKT1*;*5*, and *OsAPX2* genes were strongly impaired in shoots of two *OeJcERF011* lines under 150 mM NaCl treatment. In addition, the expression levels of 3 salt stress-related transcription factor genes, *OsDREB1A*, *OsDREB2A* and *SNAC1* (*STRESS-RESPONSIVE NAC 1*) [66, 67], were about 2-fold lower in the *OeJcERF011* shoots than in wild-type shoots under this salinity stress condition.

## Discussion

In a previous study it was hypothesized that horizontal transfer of an HNH-AP2 endonuclease from bacteria or viruses into plants may have given rise to the AP2/ERF family of transcription factors via transposition and homing processes [5]. During the evolution of plants, tandem duplication events affecting members of the HNH-AP2 family resulted in genes containing two or more AP2 domains, and in higher plants, so far as we know at present, only the genes containing two AP2 domains remain in their genomes. In this study, we identified a putatively complete set of *AP2/ERF* genes in the physic nut genome, comprising a total of 16 AP2, 4 RAV, 1 Soloist, and 98 ERF encoding genes. As reported by Shigyo *et al* [18], the evolution of the plant AP2/ERF superfamily, to produce the AP2 family (divided into AP2 and ANT subfamilies), the ERF family (divided into ERF and DREB subfamilies), and the RAV and Soloist genes, took place following the divergence of the land plant lineage from the green algae, based on phylogenetic analysis and gene structures (S2 Fig). Unlike Arabidopsis, physic nut has a single AP2/ERF domain containing protein assigned to the ANTc subfamily but no genes assigned to the AP2 subfamily (S3 Table). In the ERF family, physic nut lacks genes of the Xb-L subgroup, which exist in *Populus trichocarpa* [12] and *Citrus sinensis* [68]. Physic nut and the other dicots tested in this study had no group XI to XIV genes, which are present in rice [7]. These results indicate that different plant lineages may have either lost or acquired some of the AP2/ERF subfamilies during their evolution. In addition, the *JcERF043* and *JcERF071* genes contain nonsense mutations (S1 Fig), suggesting that they are nonfunctional in the physic nut genome.

In physic nut, all genes in the AP2 family and the Soloist family have introns, as is the case in many other plants including moss (*P*. *patens*) (S2 Fig). The conserved intron sites within each subfamily imply that the introns evolved before gene duplication in each subfamily. Only some of the ERF subfamily genes (subgroups Va, VIIa, and Xa/c) (22.2% and 27.7% in physic nut and Arabidopsis, respectively) and few if any DREB genes (0 and 1 gene in physic nut and Arabidopsis, respectively) were found to contain introns within their ORFs. However, in *P*. *patens*, 29 ERF (61.7%) and 19 DREB (34.0%) genes contain introns. The exon—intron structures of *P*. *patens* ERF genes were classified into 5 models, four of which (models 1–4) had introns in different sites of the AP2/ERF domain coding sequences. A total of 19 ERF and 10 DREB genes belong to the same model, model 1 (Fig 2). The intron in the subgroup Va genes of higher plants belongs to model 4. Fewer members of the ERF gene family, especially the DREB genes, contain introns in higher plants than in *P*. *patens*, implying that introns were probably lost during the evolution of higher plants.

Gene duplication has occurred throughout plant evolution, contributing to the establishment of new gene functions, and underlying the origins of evolutionary novelty [54, 69]. According to whole genome analysis of the *AP2/ERF* genes in Arabidopsis [7], *Populus* [12], *V*. *vinifera* [9], and rice [7], multiple segmental and tandem duplication events played important roles in the elaboration of the *AP2/ERF* gene family. Four potential *AP2/ERF* genes containing chromosomal/segmental duplications were detected in the physic nut genome, indicating that there was an ancient duplication event (Fig 3). Fifteen tandem arrays were identified (Fig 3), all of which were also detected in genomes of monocotyledonous and/or dicotyledonous species (S3B Table). These results suggest that the expansion of the *AP2/ERF* gene family in physic nut resulted from ancient duplication events which occurred both before and after the separation of the monocot and dicot lineages. Six ERF family tandem duplicates present in both monocots and dicots could be divided into different subgroups (S3B Table), suggesting that their sequence divergence occurred before the separation of monocots and dicots. In physic nut, several duplicates (in A2, A3, A4, T3, T11, T12, and T15) show divergent patterns of expression (S4 Table), suggesting the occurrence of subfunctionalization during the evolutionary process.

The gene *JcERF011* belongs to the subgroup IIIe of ERF family. Overexpression of *JcERF011* (*OeJcERF011*) in Arabidopsis results in a dwarf phenotype (S3 Fig), an effect similar to that of an Arabidopsis subgroup IIIe gene, At5g25810, which plays a negative role in regulating cell expansion [70, 71]. However, *OeJcERF011* rice plants show a normal phenotype under normal growth condition (Fig 6A and 6C). These results support the hypothesis that the different DREB-like transcription factors or the same DREB transcription factors but in different transgenic plant backgrounds, may make different contributions to plant growth processes [72].

A large number of studies have reported that many DREB genes were involved in abiotic stress responses and overexpression of DREB genes could enhance their tolerance to abiotic stress in plants [73]. In this study, we observed that the expression of *JcERF011* was downregulated by salt stress treatment in physic nut roots (Fig 5B). Overexpressing the *JcERF011* gene in rice enhanced its sensitivity to salinity stress including stronger growth inhibition of plants (Fig 6C) and higher relative electrolyte leakage from leaves (Fig 6D). The higher relative electrolyte leakage indicated greater cell membrane damage of *OeJcERF011* leave cells than wild-type leave cells under these salt stress conditions. In order to dissect the reduced salt tolerance at the molecular level, expression of 8 salt stress-responsive genes were monitored between the transgenic and wild-type rice seedlings. Our results showed that seven out of eight were significantly lower expression in transgenic rice compared to wild-type (Fig 6E). These results suggested that *JcERF011* might be involved in many pathways of the salt response in the transgenic rice plants. A lot of efforts are still required to uncover in detail of the regulation of the salt stress-responsive genes in rice, and the function of the other two subgroup IIIe genes from physic nut and subgroup IIIe genes from other plant species.

## Conclusion

A total of 119 *JcAP2/ERF* genes were identified in the physic nut genome. Duplications of the *JcAP2/ERF* genes in physic nut arose from ancient duplication events. The abundances of *JcAP2/ERF* gene transcripts were measured in different tissues under normal growing conditions. Thirty-eight *JcAP2/ERF* genes responded to abiotic stressors. Overexpression of *JcERF011* gene in Arabidopsis and rice resulted in different biological effects in transgenic plants of the two species. The overexpressed *JcERF011* gene has a negative effect on salt tolerance in rice. The results of the analyses presented here will make it possible to design additional experiments to investigate the functional conservation of *AP2/ERF* genes and determine their precise roles in development and stress responses in different plant lineages.

## Supporting Information

S1 Fig Putative nofunctionalization nature of *JcERF043* and *JcERF071* genes in the physic nut genome. (PDF)

S2 Fig Maximum likelihood phylogenetic trees of AP2/ERF proteins and alignment of the amino acids of R1 and R2 repeats in representative AP2/ERF genes. (PDF)

S3 Fig Overexpression of *JcERF011* caused dwarf phenotype in Arabidopsis. (PDF)

S1 Table Primers used in this study. (XLSX)

S2 Table Overview of the *AP2/ERF* genes detected in the physic nut genome. (XLSX)

S3 Table Orthologous groups of *AP2/ERF* genes from physic nut and six other species. (XLSX)

S4 Table The expression of *JcAP2/ERF* genes based on digital gene expression analysis. (XLSX)
