# Supplementary material for: Genome-Wide Analysis of the AP2/ERF Gene Family in Physic Nut and Overexpression of the JcERF011 Gene in Rice Increased Its Sensitivity to Salinity Stress
Source: PLoS One. 2016 Mar 4;11(3):e0150879. doi: 10.1371/journal.pone.0150879 (PMC4778941; doi:10.1371/journal.pone.0150879)
Supplement: S1 Fig — (PDF) [file pone.0150879.s001.pdf]

S1 Fig. Putative nofunctionalization nature of *JcERF043* and *JcERF071* genes in the physic nut genome.

```
JcERF043        ATGTCATTGCCACAACTTACCCAAGATCAAGAACTCTCCATCATGGTCTCTGCTCTGAAA 60
JiERF043        ATGTCATTGCCACAACTTGCCCAAGATCAAGAGCTCTCCATCATGGTCTCTGCTCTGAAA 60
                ****************** ************* ***************************

JcERF043        AATGTTGTCTCCGGCGACGCCACCAGTAGCACAACGGTGGCTTCAGATTTCTCACAGGTT 120
JiERF043        AATGTTGTCTCCGGCGACGCCACCAGTAGCACAACGGTGGCTTCAAATTTCTCACAGGTT 120
                ********************************************* **************

JcERF043        CGTCAATTCCCCTCCAATGGTTATGGTTATAGTTCTAGTTACTCCTCTGCCGCTGTTACT 180
JiERF043        CGTCAATTCCCCTCCATTGGTTATGGTTATAGCTCTAGCTCCTCCGCTGCCGTTGTTACT 180
                **************** *************** ***** * **** ****** *******

JcERF043        GGTGCAGTTGCTACCACCACCACTAGTTTTTATAACGCATCAGATCTTGACACGTGTAGC 240
JiERF043        GGTGCAGTTGCTACCACCACCGCTAGTTTTTATAACGCATCAGATCTTGACACGTGTAGC 240
                ********************* **************************************

JcERF043        ATATGCAAAATCAACGGATGTTTAGGATGCAACTTCTTCCCGCCAAATCAAGAGGAAAAA 300
JiERF043        ATATGCAAAATCAACGGATGTTTAGGATGCAACTTTTTCCCTCCAAATCAAGAGGAAAAA 300
                *********************************** ***** ******************

JcERF043        AAGGGGAGAAGAAAAAGAATAAAGAAAAATTACAGAGGAGTAAGGCAGAGGCCGTGGGGA 360
JiERF043        AAGGGGAGAAGAAAAAGAATAAAGAAAAATTACAGAGGAGTAAGGCAGAGGCCGTGGGGA 360
                ************************************************************

JcERF043        AAATGGGCGGCGGAGATAAGAGACCCACGGCGAGCGACAAGGGTGTGGCTAGGGACTTTT 420
JiERF043        AAATGGGTGGCGGAGATAAGAGACCCACGGCGAGCGACAAGGGTGTGGCTAGGGACTTTT 420
                ******* ****************************************************
                            Stop
JcERF043        AACACGGCGGTGTAGGCTGCTAGAGCCTATGATAAGGCCGCGATAGATTTTCGAGGGCCG 480
JiERF043        AACACGGCGGAGGACGCTGCTAGAGCCTATGATAAGGCGGCGATAGATTTTCGAGGGCCG 480
                ********** * * *********************** *********************

JcERF043        AGAGCGAAGCTGAATTTTCCATTTCCAGATAATACAGACAGTAATGGTTCAAAATTAGGA 540
JiERF043        AGAGCCAAGCTGAATTTTCCATTTCCAGATAATACAGACAGTAATGGTTCGAATTTAGGA 540
                ***** ******************************************** ** ******

JcERF043        GAGCAAATTATTCCTCAAGAACAAGAACAGAAAGAAAATAGGAAAGGAAAGGAATTTGAA 600
JiERF043        GAACAAATTATTGCTCAAGAACAAGAACAGAAAGAAAATAGGAAAGGAAAGGAATTTGAA 600
                ** ********* ***********************************************

JcERF043        GTGGAAATGGAAAATCGAAACGATTTTGAATTCTGGGAAGGAGTTGGTAAAGATGAAATT 660
JiERF043        GTGGAAATGGAAAATCGAAAGGATTTTGAATTCTGGGAAGGAATTGGTAAAGATGAAATT 660
                ******************** ********************* *****************

JcERF043        GAACAATGGA---------TGATGAATTTTAATAGGGATTCATCAGATTCTTCTGCTACT 711
JiERF043        GAACAATGGACGATGATGATGATGAATTTTAATGGGGATTCATCAGATTCTTCTGCTACT 720
                **********         ************** **************************

JcERF043        ATTATTTTGTAA 723
JiERF043        ATTAATTTGTAA 732
                **** *******
```

(A), DNA sequence alignment of ERF043 coding domain sequences from *J. curcas* and *J. integerrima*, showing a nonsense mutation site in *JcERF043*.

S1 Fig. (Continued.)

```
JcERF042        MHKSSKRLKHTGPPPTEYLPPAMLLPRLTQDQELFIMVSALKNVVSG-TTSSTSVATDL- 58
JiERF042        MHKSSKRLKHTGPPPTEYLPPVMPLPRLTQDQELSIMVSALKNVVSG-TTSSTSVATDL- 58
JcERF043        ----------------------MSLPQLTQDQELSIMVSALKNVVSGDATSSTTVASDFS 38
JiERF043        ----------------------MSLPQLAQDQELSIMVSALKNVVSGDATSSTTVASNFS 38
                                      * **:*:***** ************ :****:**:::

JcERF042        -FHQFAAAAS--SSSPATAVASTAVTTTTDSLYNPIPPPSDLDTCNICKIKGCLGCNFFP 115
JiERF042        -FHQFTSAAS--SSSSATAVASTAVSTTTASLYNPIPPPSDLDTCNICKIKGCLGCNFFP 115
JcERF043        QVRQFPSNGYGYSSSYSSAAVTGAVATTTTSFYN----ASDLDTCSICKINGCLGCNFFP 94
JiERF043        QVRQFPSIGYGYSSSSSAAVVTGAVATTTASFYN----ASDLDTCSICKINGCLGCNFFP 94
                 .:**.: .   *** ::*..: **:.** *:**    .******.:***:*********

                                              AP2/ERF domain
JcERF042        PNQEEKKGTRKRVKKNYRGVRQRPWGKWAAEIRDPRRATRVWLGTFNTAEEAARAYDKAA 175
JiERF042        PNQEEKKGTRKRVKKNYRGVRQRPWGKWAAEIRDPRRATRVWLGTFNTAEEAARAYDKAA 175
JcERF043        PNQEEKKGRRKRIKKNYRGVRQRPWGKWAAEIRDPRRATRVWLGTFNTAV•AARAYDKAA 153
JiERF043        PNQEEKKGRRKRIKKNYRGVRQRPWGKWVAEIRDPRRATRVWLGTFNTAEDAARAYDKAA 154
                ******** ***:***************.********************  *********

JcERF042        IDFRGPRAKLNFPFPDNTAS-----NLEGQSTPQEQQQKENGKRKELEVEMQVGKEYEFW 230
JiERF042        IDFRGPRAKLNFPFPDNTASASNITNLEGQSTPQEQQQKENGKRK----DMQLGNEHEFW 231
JcERF043        IDFRGPRAKLNFPFPDNTDS--NGSKLGEQIIPQEQEQKENRKGKEFEVEMENRNDFEFW 211
JiERF043        IDFRGPRAKLNFPFPDNTDS--NGSNLGEQIIAQEQEQKENRKGKEFEVEMENRKDFEFW 212
                ****************** *     :*  *  .***:**** * *    :*:  ::.***

JcERF042        EKIGEDEIQQWMN-MMDFNGDSSDSTGTAATTTIAN 265
JiERF042        EKIGEDEIQQWMN-MMDFNGDSSDSTGTA--TTIAN 264
JcERF043        EGVGKDEIEQW---MMNFNRDSSDSSATIIL----- 239
JiERF043        EGIGKDEIEQWTMMMMNFNGDSSDSSATINL----- 243
                * :*:***:**   **:** *****:.*
```

(B), Amino acid sequence alignment of ERF042 and ERF043 proteins from *J. curcas* and *J. integerrima*: the nonsense mutation in the *JcERF043* gene results in an incomplete AP2/ERF domain in the protein that it encodes.

S1 Fig. (Continued.)

```
JcERF071        --------------MHSLASPIMQPL---------------------------------- 12
JcERF070        MEAETHVASALEFIKHHLLGDLLSPVGASCSYSTLNDVNMELSSSENESNCSRTSSIDST 60
                               * * . ::.*:

JcERF071        -------------------------------------------ALPASLTQSSRSFLQP- 28
JcERF070        ISFSDYFTSNNIDDDFFQFEAKPQIVDLTAPRSCDSPTSFEFDAKPQLISQSSNEFFEFE 120
                                                           * *  ::***..*::

JcERF071        ------------------------------------------------------------
JcERF070        SKPQIQHIQNFRFESKPQSISLSNNVFNDFESKPEIPSVGSSSQGNRKPSLKISLPKKTE 180

                                           AP2/ERF domain
JcERF071        -----------VQADSGAFAVEKKRHYGGVRQRPWSKYAAEIRDPNRKGRRLWLGTFD-- 75
JcERF070        WIQFAEPNQQSVQADSGASAVEEKRHYRGVRQRPWGKYAAEIRDPNRKGTRLWLGTFETA 240
                           ******* ***:**** *******.************* *******:

JcERF071        -EAAKAYDQAAFKFCGSKAILNFTLKARKWNTRSSEASEQKGTGESEIVEEEAKKAVRER 134
JcERF070        IEAAKAYDQAAFKFRGSKAILNFPLEAGKWNTRASEGNERKRTRESEIVEEEAKKVVK-- 298
                 ************* ********.*:* *****:**..*:* * ***********.*:

JcERF071        SLREMWRCRRRAGRLSWTGRVALG--LGILGEKKLG--AQNGEYGRFDTLGLG 183
JcERF070        --REEPQGDEALTPSSWTAIMDGGDLKGIFNVPPLSPFSPHPPLG-YSQLIVS 348
                  **  :  .     ***. :  *   **:.   *.  : :   * :. * :.
```

(C), Amino acid sequence alignment of annotated JcERF071 and JcERF070 proteins. JcERF071 has lost 3 amino acid residues from within the AP2/ERF domain.

S1 Fig. (Continued.)

```
JcERF070        ATGGAGGCTGAAA--CTCATGTAGCTTCTGCTTTAGAGTTCATCAAGCATCATTTACTTG 58
JcERF071        -TCCAAATTGAAAATTTTATATTATTATCGCCCTAAA----TTCAAAT----TTTGCTAA 51
                 *  *   *****   * ** *   *   **  ** *     ****      *** **

JcERF070        GTGATCTTCTCTCACC--TGTTGGTGCTTCATGTTCTTATTCCACCCTTAATGATGTAAA 116
JcERF071        G--ATTTTAAATAAAAAATATTAAAGTG----GATCCCACAGCACCCCTT-TGGGGTTAA 104
                *  ** **   * *    * **   *      * **  *   ***** *  **  ** **

JcERF070        TATGGAGCTTTCGAGCTCTGAAAATGAGTC-TAATTGCTCCCGGACTTCAAGTATTGATT 175
JcERF071        CTTAA-----CCAAGGTGTCAAAAGGCGTCGTTTTTGACACCATACTC--ACCATT--TT 155
                  *        * ** * * **** * *** *  ***   **  ***   *  ***  **

JcERF070        CAACAATCTCATTCTCTGATTACTTTACAAGCAACAATATTGATGACGATTTCTTCCAAT 235
JcERF071        CAACAATCTCAT-CTTCAATCACTGTCT--TCTTGAATGCCAAAGA--ACCCCTAC---- 206
                ************ **   ** *** *     *   ***    * **  *   ** *

JcERF070        TTGAAGCTAAACCTCAAATCGTTGATCTCACTGCTCCAAGATCTTGCGATTCGCCAACTA 295
JcERF071        -----GC--AACCTCAAATCGTCGATCTCACTGCTCCAAGATCTTGTGATTCGTCAACCA 259
                     **  ************* *********************** ****** **** *

JcERF070        GTTTTGAATTTGATGCAAAACCTCAACTAATCTCTCAATCAAGTAACGAATTCTTCGAAT 355
JcERF071        GTTTTGAATTTGATGCAAAACCTCAACTAATCTCTCAATCAAGTAACGAAATCTTCGAAT 319
                ************************************************** *********

JcERF070        TCGAATCAAAACCTCAAATACAACACATTCAGAATTTCAGATTTGAATCAAAA-CCTCAA 414
JcERF071        TCGAATCAAAACCTCAAATACAACACATTCAGAATTTCCGATTTGAATCAAAAACCTCAA 379
                ************************************** ************** ******

JcERF070        AGTATTTCTCTATCAAATAACGTTTTCAACGATTTCGAATCAAAACCTGAAATACCATCT 474
JcERF071        AGTATTTCTCAATCAAATAACGTTTTCAATGATTTTGAATCAAAACCTCAAATATCACCG 439
                ********** ****************** ***** ************ ***** ** *

JcERF070        GTTGGTTCAAGTTCTCAGGGGAACCGAAAGCCGTCTTTGAAAATATCGTTGCCCAAAAAA 534
JcERF071        GTTGGTTCAACTTCTACGACGAATTAAAAGTCGTACTTCAAGATA------CTCA----- 488
                ********** ****  *  ***   **** ***  ** ** ***      * **
                                        Stop

JcERF070        ACCGAATGGATCCAATTTGCTGAACCGAACCAGCAATCGGTCCAAGCTGACTCGGGTGCT 594
JcERF071        ----------TCCAATTTGCTGAACGGAACCAGCAACCGGTCCAAGCTGACTCGGGTGCT 538
                          *************** ********** ***********************

JcERF070        TCTGCTGTAGAAGAGAAAAGGCATTACAGAGGAGTCCGACAGAGACCGTGGGGCAAATAC 654
JcERF071        TTTGCTGTAGAAAAGAAAAGGCATTACGGAGGAGTCCGACAGAGACCGTGGAGCAAATAC 598
                * ********** ************** *********************** ********

JcERF070        GCAGCGGAGATCCGAGACCCGAACCGGAAAGGCACTCGGTTGTGGCTCGGAACCTTCGAA 714
JcERF071        GCAGCGGAGATCCGAGACCCGAATCGGAAAGGCCGTCGATTGTGGCTCGGCACATTCGA- 657
                *********************** *********  *** *********** ** *****

JcERF070        ACAGCTATCGAAGCCGCCAAAGCCTACGATCAAGCAGCGTTCAAGTTTCGCGGCTCAAAA 774
JcERF071        --------CGAAGCCGCCAAAGCCTACGATCAAGCAGCGTTCAAGTTTTGCGGCTCAAAA 709
                        **************************************** ***********

JcERF070        GCCATTCTGAACTTCCCTCTAGAAGCTGGGAAATGGAACACACGTGCCAGTGAAGGAAAC 834
JcERF071        GCCATTCTGAACTTCACTCTAAAAGCTAGGAAATGGAACACACGTTCCAGTGAAGCAAGC 769
                *************** ***** ***** ***************** ********* ** *

JcERF070        GAACGGAAACGAACAAGAGAAAGTGAGATTGTAGAAGAGGAAGCGAAGAAGGTGGTGAAG 894
JcERF071        GAACAGAAAGGAACAGGAGAAAGTGAGATTGTAGAAGAGGAAGCGAAGAAGGCGGTGA-G 828
                **** **** ***** ************************************ ***** *

JcERF070        AGAGAAGAGCCTCAGGGAGATGAGGCGTTGACGCCGTCGAGCTGGACGGCAATTATGGAC 954
JcERF071        AGAGAGGAGCCTACGAGAGATGTGGCGTTGTCGCCGTCGAGCTGGACGGCTATCATGGAC 888
                ***** ******  * ****** ******* ******************* ** ******

JcERF070        GGAGG----CGATTTGAAA---GGAATCTTC----------AAT---------------- 981
JcERF071        GGGGAGAGTTGCTTTAGGATTAGGGATTTTGGGGAAAAAAAAATTGGGGGCGCAAAACGG 948
                ** *      * ***   *   ** ** **           ***

JcERF070        ---GTACCGCCGTTGT-----CTCCGTTTT--------CTCCTC---------------- 1009
JcERF071        TGAGTATGGTCGTTTTGACACCTTGGGCTTGGGTTAAGCTGATCCAACAGTGGTGCTATG 1008
                   ***  * **** *     **  *  **        **  **

JcERF070        --ATCCGCCGTTAGGATATTCTCAGCTTATAGTTTCA-TAA--------- 1047
JcERF071        GGATCCACT-TTAATATTTTTTTA-TTTAAAATTCTAGCAAAATTTGAAT 1056
                  **** *  ***  ** ** * *  *** * **  *  **
```

(D), DNA sequence alignment of *JcERF071* and *JcERF070* genes, showing DNA sequence divergence.
