# Supplementary material for: Genome-Wide Analysis of the AP2/ERF Gene Family in Physic Nut and Overexpression of the JcERF011 Gene in Rice Increased Its Sensitivity to Salinity Stress
Source: PLoS One. 2016 Mar 4;11(3):e0150879. doi: 10.1371/journal.pone.0150879 (PMC4778941; doi:10.1371/journal.pone.0150879)
Supplement: S2 Fig — (PDF) [file pone.0150879.s002.pdf]

S2 Fig. Maximum likelihood phylogenetic trees of AP2/ERF proteins and alignment of the amino acids of R1 and R1 repeats in representative *AP2/ERF* genes. Phylogenetic analysis was conducted using AP2/ERF domain protein sequences. Branch support values correspond to approximate likelihood ratio test (aLRT) results. Bootstrap scores higher than 50% are indicated on the nodes. Distinct orthologous groups (subgroups) are indicated. Detailed information about the groups is given in Table S3.

(A), Maximum likelihood phylogenetic tree of families AP2, RAV, and Soloist from physic nut (Jc), Arabidopsis (A), rice (O), *Physcomitrella patens* (P), and *Chlamydomonas reinhardtii* (C). (B), Alignment of the amino acids of R1 and R2 repeats in representative AP2 genes, indicating divergence of the *Physcomitrella patens* AP2 R2 domain from those in other species.

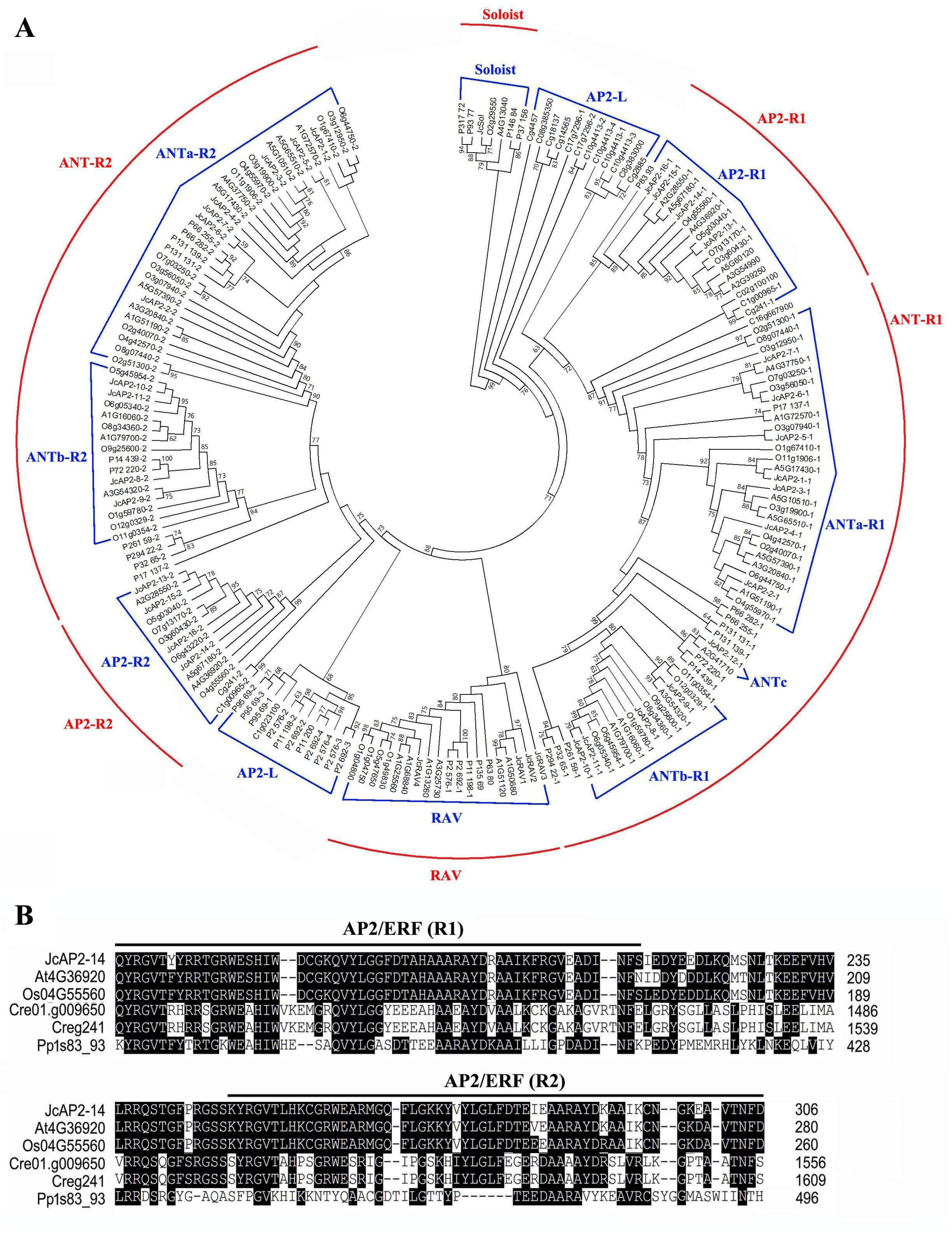

S2 Fig. (Continued.)

C

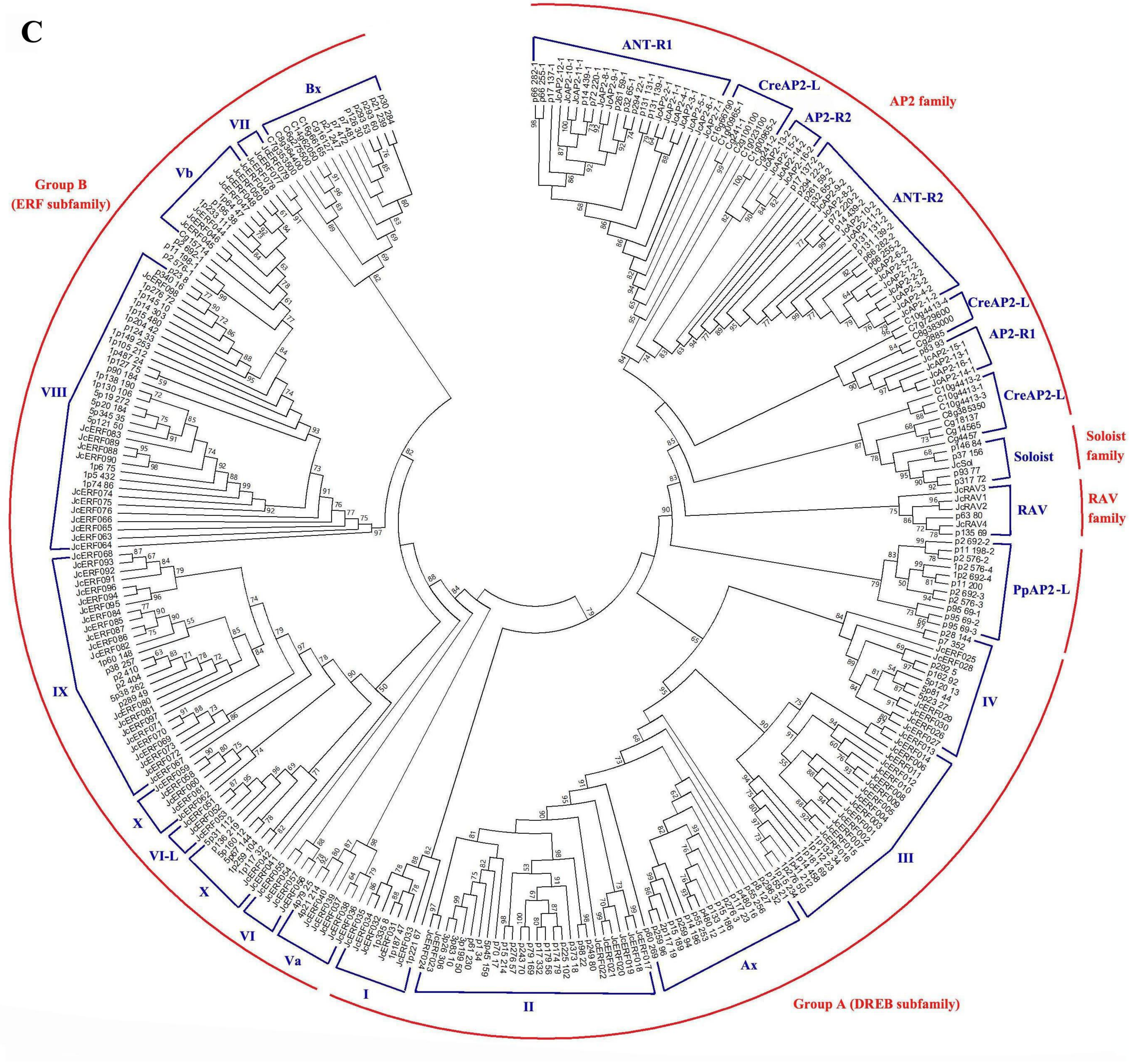

(C), Maximum likelihood phylogenetic tree of AP2/ERF proteins from physic nut (Jc), *Physcomitrella patens* (p), and *Chlamydomonas reinhardtii* (C).

S2 Fig. (Continued.)

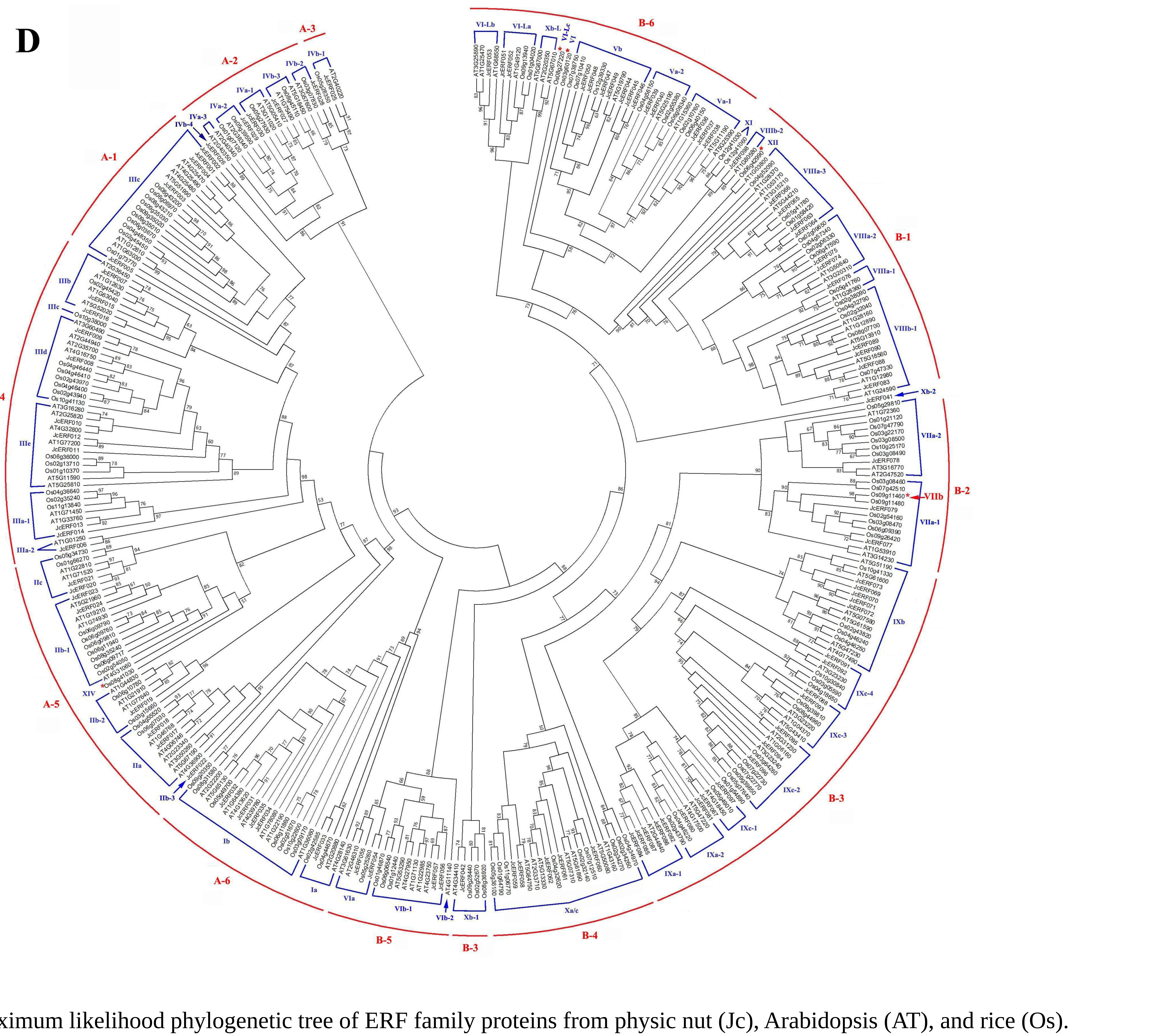

(D), Maximum likelihood phylogenetic tree of ERF family proteins from physic nut (Jc), Arabidopsis (AT), and rice (Os).

S2 Fig. (Continued.)

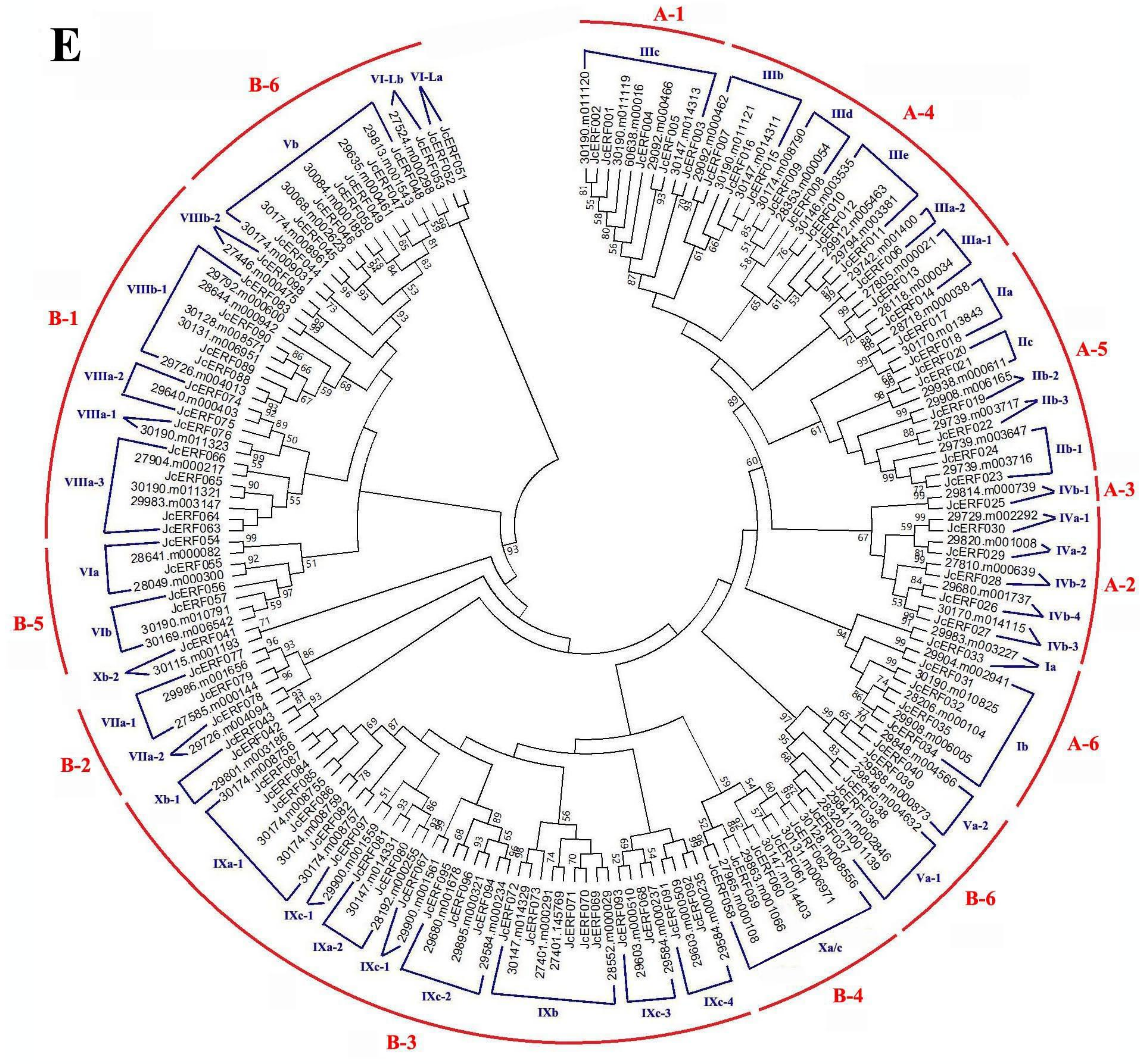

(E), Maximum likelihood phylogenetic tree of ERF family proteins from physic nut (Jc) and castor bean.

S2 Fig. (Continued.)

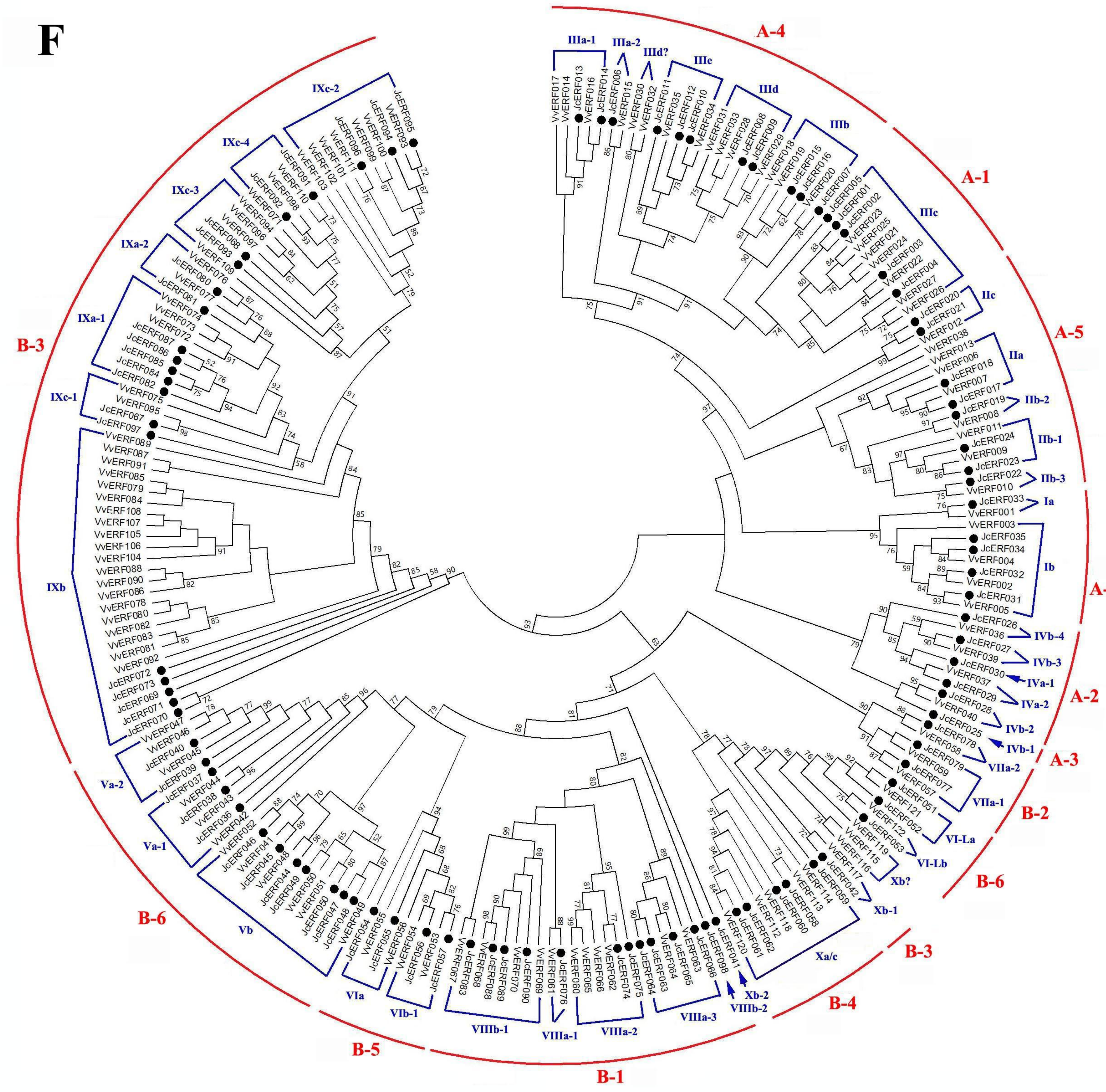

(F), Maximum likelihood phylogenetic tree of ERF family proteins from physic nut (Jc) and *Vitis vinifera* (Vv).
