# Supplementary figures and images for: Genome-Wide Analysis of the AP2/ERF Gene Family in Physic Nut and Overexpression of the JcERF011 Gene in Rice Increased Its Sensitivity to Salinity Stress
Source: PLoS One. 2016 Mar 4;11(3):e0150879. doi: 10.1371/journal.pone.0150879 (PMC4778941; doi:10.1371/journal.pone.0150879)

**S3 Fig. Overexpression of *JcERF011* caused dwarf phenotype in Arabidopsis.**

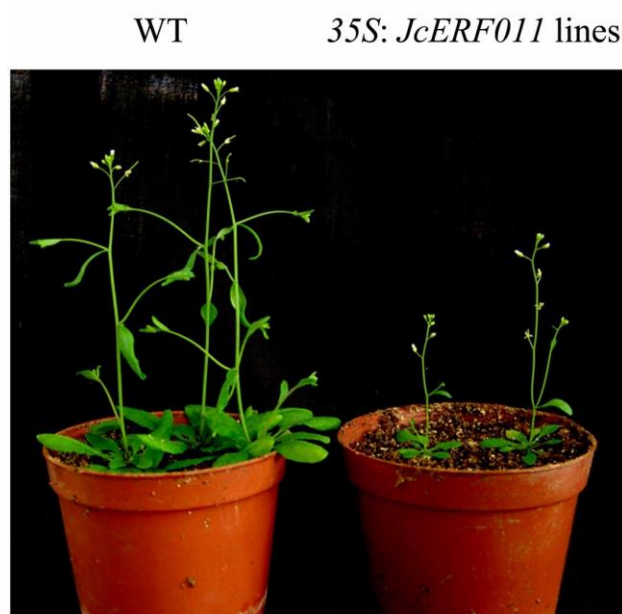

Supplement: S3 Fig — (PDF) [file pone.0150879.s003.pdf]
